# Impacts of climate change on mental health and its underlying mechanisms: an umberella review

**DOI:** 10.1186/s12991-026-00668-z

**Published:** 2026-05-13

**Authors:** Arezoo Davariniamotlaghquchan, Zeinab Niazmand, Mohsen Shafiee, Abbas Ostadtaghizadeh

**Affiliations:** 1 Department of Health in Emergencies and Disasters, School of Public Health, Tehran University of Medical Sciences, Tehran, Iran; 2 Climate Change and Health Research Center (CCHRC), Institute for Environmental Research, Tehran University of Medical Sciences, Tehran, Iran; 3 Department of Nursing, Abadan University of Medical Sciences, Abadan, Iran

**Keywords:** Climate change, Mental health, Umberella review

## Abstract

**Background:**

Climate change represents a major global health challenge with potential implications for mental health. Exposure to climate-related stressors is associated with an elevated risk of psychiatric disorders, including trauma- and stressor-related disorders (e.g., PTSD), depressive disorders, and anxiety disorders. Vulnerable populations—including children, women, older adults, individuals with pre-existing mental health conditions, and communities in low-income or disaster-prone regions—may be disproportionately affected. This umbrella review synthesizes current evidence on the mental health impacts of climate change, focusing on clinically relevant outcomes and underlying mechanisms.

**Methods:**

A systematic literature search was conducted across Web of Science, PubMed, Scopus, and Google Scholar for systematic reviews published between January 2014 and October 2024. Data extraction and methodological quality assessment were performed using the Joanna Briggs Institute Critical Appraisal Checklist, which evaluates methodological rigor, clarity of research questions, and appropriateness of data synthesis. Only English-language systematic reviews scoring ≥ 5/11 on the JBI checklist—reflecting moderate to high methodological quality—were included. Non-systematic reviews and studies without accessible full texts were excluded. The review protocol was registered in PROSPERO (CRD420251133963).

**Results:**

Climate change may affect mental health through both direct and indirect pathways. Direct impacts include elevated risk or worsening of PTSD, depressive disorders, anxiety disorders, and suicidal behaviors, which may be precipitated or exacerbated by climate-related stressors. Indirect effects operate via socioeconomic disruptions, such as food insecurity, forced migration, poverty, and weakened social networks. Psychological responses described as eco-anxiety and solastalgia further illustrate the range of mental health outcomes associated with environmental changes.

**Conclusion:**

Climate change is associated with clinically relevant psychiatric outcomes across established diagnostic categories. The mechanisms underlying these associations involve complex neurobiological, socioeconomic, environmental, and cultural pathways. These findings underscore the importance of targeted psychiatric interventions, including cognitive behavioral therapy, trauma focused therapies, resilience-building, strengthening social support, promoting adaptive coping strategies, and enhancing preparedness of mental health services. Prioritizing vulnerable populations for psychiatric assessment, prevention, and intervention is essential. Integrating these strategies into clinical practice and public health planning is critical to support evidence-based mental health care.

## Introduction

Anthropogenic climate change has emerged as a defining global health challenge, largely attributed to greenhouse gas emissions associated with fossil fuel combustion and large-scale environmental modification [1, 2]. Contemporary climate projections indicate that these processes are widely projected to intensify, contributing to increased frequency and severity of extreme weather events, ecological disruptions, and environmental instability [3–5]. These climatic shifts are associated with broad environmental transformations, including changes in precipitation patterns, rising temperatures, increased air pollution, ecosystem degradation, and biodiversity loss [6–9]. These transformations have substantial implications for human health. Climate change influences health through multiple interacting pathways, encompassing both direct effects (e.g., heat exposure, extreme weather events) and indirect mechanisms (e.g., food insecurity, altered vector ecology, water quality deterioration, and socioeconomic disruption) [10, 11]. The cumulative nature of these exposures presents complex challenges for health systems, particularly in low- and middle-income countries where adaptive capacity may be constrained [11, 12]. Mental health represents a critical yet comparatively under-integrated dimension of climate-related health research. Disturbances in psychological well-being are associated with significant functional, social, and physiological consequences, underscoring the importance of examining mental health within broader public health frameworks [13]. Evidence from epidemiological and clinical studies suggests that climate-related stressors may be associated with a range of mental health outcomes, including anxiety, depression, post-traumatic stress disorder (PTSD), cognitive disturbances, substance use, and psychological distress, particularly among populations experiencing heightened exposure or vulnerability [14–23]. Importantly, the mental health consequences of climate change extend beyond immediate psychological responses. Acute climate events may precipitate short-term psychiatric manifestations, whereas chronic environmental stressors—such as sustained temperature increases, prolonged droughts, and ecological degradation—may contribute to longer-term mental health and psychosocial risks. These processes may operate through mechanisms including economic instability, livelihood disruption, displacement, social fragmentation, and identity-related stressors [24–26]. Although there is a growing body of research examining the interplay between climate change and mental health, there is a notable absence of integrative reviews that simultaneously evaluate neurobiological, socioeconomic, environmental, and cultural mechanisms. The current literature predominantly emphasizes isolated dimensions, thereby hindering the development of comprehensive prevention and adaptation strategies. Notably, women and other socially marginalized groups are subject to distinct exposures and vulnerabilities exacerbated by structural inequalities, gender norms, and limited resource access. Unfortunately, their specific risks often remain underrepresented in the discourse. This umbrella review aims to address these deficiencies by providing a consolidated synthesis focused on mechanisms, emphasizing population-specific susceptibilities, and delivering insights to inform public health policy, climate adaptation strategies, and targeted mental health interventions. Despite a rapidly expanding literature, important conceptual and methodological gaps remain. Prior reviews have predominantly emphasized prevalence estimates, risk factors, or specific exposures, while comparatively limited attention has been directed toward integrative syntheses that explicitly examine mechanistic pathways linking climate-related exposures to psychiatric outcomes. Clarifying these mechanisms is essential for advancing theory, and informing prevention and intervention strategies within psychiatric and mental health systems.

## Methods

### Study design

This umbrellareview synthesized existing systematic reviews to evaluate the impact of climate change on mental health, encompassing a broad spectrum of psychiatric outcomes, including anxiety, depression, trauma- and stress-related disorders, mood disorders, substance use disorders, and suicidal behaviors. The review adhered to PRISMA 2020 guidelines, and the protocol was registered in PROSPERO (CRD420251133963).

### Search strategy

A thorough review was carried out utilizing major databases, such as Web of Science, PubMed, Scopus, and the Google Scholar search engine, covering publications from January 2014 to October 2024. For Google Scholar, the first 10 pages (100 results) sorted by relevance were screened, with specific selection criteria applied for inclusion or exclusion. Exact search dates for each database were recorded to enhance reproducibility, and any updates to the searches were documented (Table 1). Keywords included “climate change,” “mental health,” “anxiety,” “depression,” and related synonyms, combined with Boolean operators and MeSH terms where applicable. The time frame aligns with the post-IPCC AR5 era, ensuring relevance to contemporary diagnostic criteria (DSM-5) and methodological standards (PRISMA 2020).

**Table 1:** serach strategy

Database/ Search engine	Search Strategy
Google scholar	(“climate change” AND “mental health” AND review*)
PubMed	(((((((((((((((“mental health“[Title/Abstract]) OR (depression[Title/Abstract])) OR (anxiety[Title/Abstract])) OR (stress[Title/Abstract])) OR (distress[Title/Abstract])) OR (psych*[Title/Abstract])) OR (“depressive symptoms“[Title/Abstract])) OR (suicid*[Title/Abstract])) OR (“quality of life“[Title/Abstract])) OR (“mental wellbeing“[Title/Abstract])) OR (PTSD[Title/Abstract])) OR (“mental disorders“[Title/Abstract])) OR (“mood disorder“[Title/Abstract])) OR (“aggressive behaviors“[Title/Abstract])) AND ((((((((((“Climate Change“[Title/Abstract])) OR (“Global Warming“[Title/Abstract])) OR (climat*[Title/Abstract])) OR (“heat wave“[Title/Abstract])) OR (“cold wave“[Title/Abstract])) OR (“sand storm“[Title/Abstract])) OR (“dust storm“[Title/Abstract])) OR (Drought[Title/Abstract])) OR (“extreme weather“[Title/Abstract]))) AND (review*[Title/Abstract])
Scopus	((TITLE-ABS-KEY ("mental health") OR TITLE-ABS-KEY (depression) OR TITLE-ABS-KEY (anxiety) OR TITLE-ABS-KEY (stress) OR TITLE-ABS-KEY (distress) OR TITLE-ABS-KEY (psych*) OR TITLE-ABS-KEY ("depressive symptoms") OR TITLE-ABS-KEY (suicid*) OR TITLE-ABS-KEY ("quality of life") OR TITLE-ABS-KEY ("mental wellbeing") OR TITLE-ABS-KEY (ptsd) OR TITLE-ABS-KEY ("mental disorders") OR TITLE-ABS-KEY ("mood disorder") OR TITLE-ABS-KEY ("aggressive behaviors"))) AND ((TITLE-ABS-KEY ("Climate Change") OR TITLE-ABS-KEY ("Global Warming") OR TITLE-ABS-KEY (climat*) OR TITLE-ABS-KEY ("heat wave") OR TITLE-ABS-KEY ("cold wave") OR TITLE-ABS-KEY ("sand storm") OR TITLE-ABS-KEY ("dust storm") OR TITLE-ABS-KEY (drought) OR TITLE-ABS-KEY ("extreme weather"))) AND (TITLE-ABS-KEY (review*))
WOS	(((((((((((((TS=(“mental health”)) OR TS=(depression)) OR TS=(anxiety)) OR TS=(stress)) OR TS=(distress)) OR TS=(psych*)) OR TS=(“depressive symptoms”)) OR TS=(suicid*)) OR TS=(“quality of life”)) OR TS=(“mental wellbeing”)) OR TS=(PTSD)) OR TS=(“mental disorders”)) OR TS=(“mood disorder”)) OR TS=(“aggressive behaviors”) AND ((((((((TS=(“Climate Change”)) OR TS=(“Global Warming”)) OR TS=(climat*)) OR TS=(“heat wave”)) OR TS=(“cold wave”)) OR TS=(“sand storm”)) OR TS=(“dust storm”)) OR TS=(Drought)) OR TS=(“extreme weather”) AND TS=(review*)

### Eligibility criteria

The inclusion criteria for this study were as follows: systematic reviews published between 2014 and 2024, written in English, and explicitly addressing the impact of climate change on mental health, particularly anxiety and depression. The exclusion criteria were: reviews that did not adhere to systematic methodology, reviews not focusing on the impact of climate change on mental health, and studies without accessible full text. Although English-language restriction was applied, this may introduce language bias, particularly affecting studies from low- and middle-income countries.

### Study selection

Two independent reviewers participated in the process of selecting studies. The initial screening of the titles and abstracts of all identified studies was performed according to the predefined inclusion and exclusion criteria. Subsequently, the titles and abstracts were independently evaluated by two authors. If any disagreements arose between the two reviewers concerning the inclusion or exclusion of a study, a third reviewer was consulted to make the final decision. This method ensured consistency and accuracy throughout the selection process.

### Data extraction

Data were methodically collected from the chosen studies using a standardized extraction template. This template encompassed essential variables, including study design, characteristics of the population, employed methodologies, key findings, and the overall quality of the study. Each of the Two reviewers was tasked with independently gathering data from a portion of the studies. Data were managed using EndNote bibliographic management software. Extracted variables included study design, population characteristics, geographical context, number of primary studies, key findings, and quality ratings. All relevant outcomes were extracted, and prioritization criteria were applied when multiple outcomes were reported. To maintain consistency, the reviewers compared the extracted data. In situations where there were discrepancies or uncertainties, discussions were held, and a final decision was reached through consensus or, when necessary, through adjudication by the third reviewer. This comprehensive data extraction process aimed to capture all pertinent information to guide the analysis and synthesis of the studies included in the review.

### Quality assessment

The methodological quality of the systematic reviews included in the analysis was assessed using the Joanna Briggs Institute (JBI) Critical Appraisal Checklist for Systematic Reviews and Research Syntheses. This evaluative tool examined adherence to established systematic review protocols, the precision of research questions, the adequacy of search strategies employed, and the robustness of data synthesis methodologies. Studies were classified into three quality categories: high (> 80%), moderate (50–79%), or low (< 50%). These quality ratings guided the interpretation and weighting of the evidence during the synthesis process.

### Risk of bias and strategies for minimization

To mitigate bias, the study employed two independent reviewers for screening and data extraction, with any discrepancies adjudicated by a third reviewer. Methodological quality and risk of bias were rigorously evaluated using the JBI checklist. To avert selective reporting, the study protocol was pre-registered in PROSPERO. Additionally, a thorough search strategy across multiple databases was implemented to incorporate both published and unpublished systematic reviews, thereby minimizing the risk of publication bias.

### Data synthesis

The findings were synthesized through an umbrella review approach. Due to methodological and population heterogeneity across the included systematic reviews, a formal meta-analysis was not conducted. Effect sizes from primary studies were not recalculated. Quantitative findings were interpreted as reported in the included reviews. Certain PRISMA items, such as individual study effect measures, were not applicable given the design of a systematic review. This approach enabled a comprehensive qualitative integration of evidence across diverse psychiatric outcomes, elucidating patterns, mechanisms linking climate change to mental health, and relevant trends while accommodating inherent study variability.

### Limitations

Although an extensive and systematic search was conducted across multiple databases, two studies that met the inclusion criteria were not accessible in full text. This limitation may have affected the comprehensiveness of the review, as the exclusion of these studies could have resulted in missing valuable data and insights. The inability to access full-text articles is a common challenge in systematic reviews, especially in fields with restricted access to certain journals or publications. This issue may have introduced a potential bias by limiting the inclusion of relevant studies, thereby potentially affecting the overall accuracy and reliability of the findings. Additionally, the studies included in the review exhibited considerable variability in terms of study populations and methodologies. Differences in the geographical locations of the studies, the populations studied (e.g., age groups, socio-economic statuses), and the methods used to measure mental health outcomes introduced heterogeneity into the synthesis process. This variability may have reduced the generalizability of the results, as it is difficult to draw broad conclusions that apply to all contexts. The diversity in study designs also made it challenging to combine the findings from individual studies, highlighting the need for more uniform methodologies in future research on the impact of climate change on mental health. Additionally, the inclusion of Google Scholar may reduce reproducibility due to its dynamic search algorithm and limited transparency in filtering and ranking results. Table 2 presents a summary of the method of this study.

**Table 2:** a summary of the method

Section	Description
Study design	Umbrella review synthesizing existing systematic reviews on the impact of climate change on mental health, including anxiety, depression, trauma- and stress-related disorders, mood disorders, substance use disorders, and suicidal behaviors; followed PRISMA 2020 guidelines; protocol registered on PROSPERO (CRD420251133963)
Databases	Web of Science, PubMed, Scopus; Google Scholar utilized as a supplementary search engine to identify additional relevant studies
Search strategy	Keywords: “climate change”, “mental health”, “anxiety”, “depression” + synonyms; Boolean operators (AND, OR) and MeSH terms used; publication period: 2014–2024
Eligibility criteria	Inclusion: systematic reviews (2014–2024), English, addressing climate change and mental health; Exclusion: non-systematic reviews, irrelevant focus, inaccessible full text
Study selection	Two independent reviewers screened titles, abstracts, and full texts; discrepancies resolved by a third reviewer; documented with PRISMA flow diagram
Data extraction	Standardized extraction form capturing study design, population characteristics, methodology, main findings, and study quality
Quality assessment	Joanna Briggs Institute (JBI) Critical Appraisal Checklist; studies categorized as high (> 80%), moderate (50–79%), or low (< 50%) quality; quality informed interpretation and weighting of evidence
Bias minimization	Independent dual screening and extraction, third-party adjudication, standardized JBI tool, PROSPERO registration, comprehensive search to minimize selection and publication bias
Data synthesis	Qualitative synthesis due to heterogeneity in populations, outcomes, and methodologies; meta-analysis not conducted; synthesis highlighted patterns, mechanistic pathways, and trends across psychiatric outcomes
Limitations	Two studies inaccessible in full text; heterogeneity in populations and methods; English-language restriction

## Results

### Results of the systematic search

**Fig. 1:**
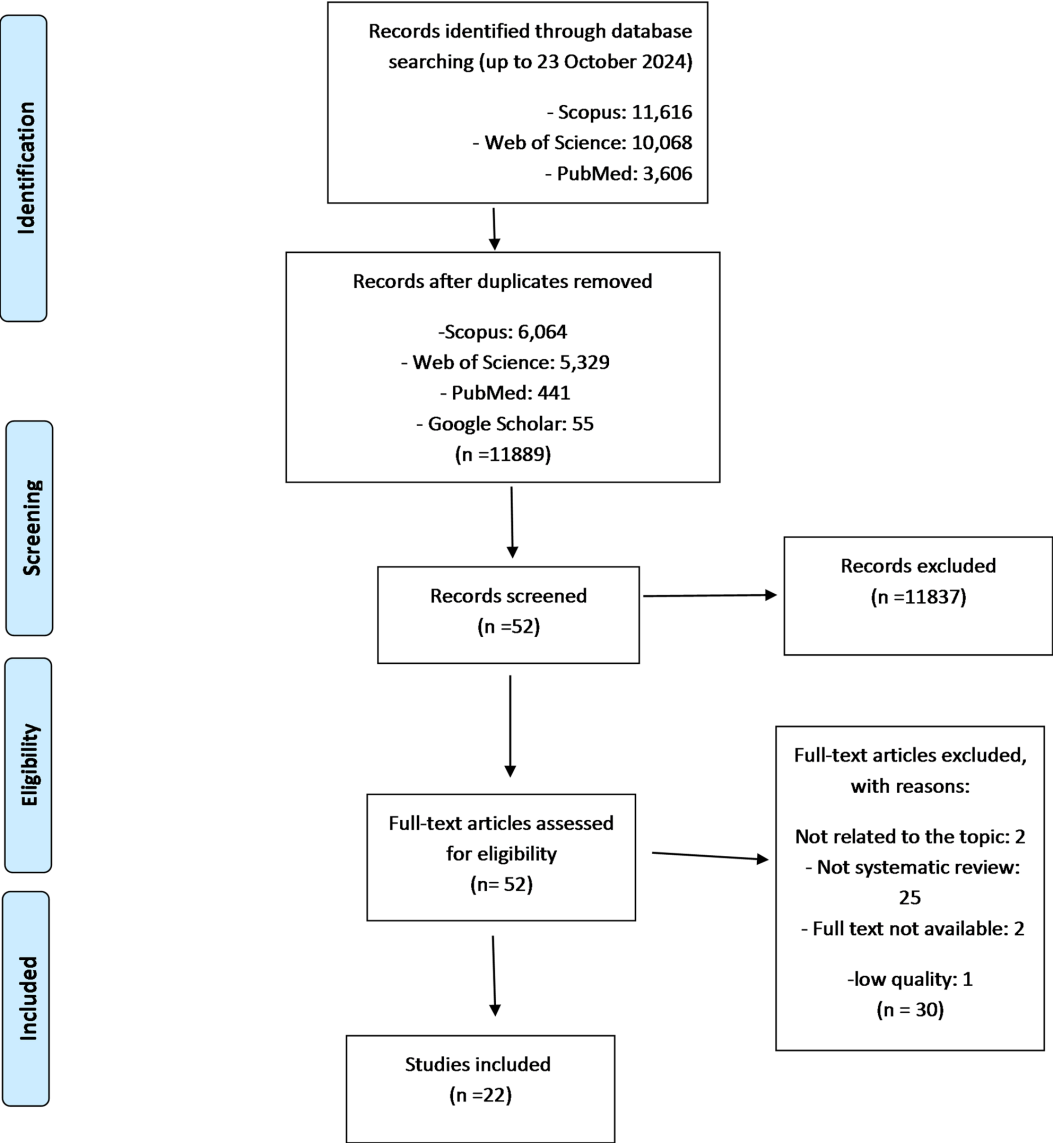
The PRISMA flow diagram for the identification of studies for this review

**Fig. 2:**
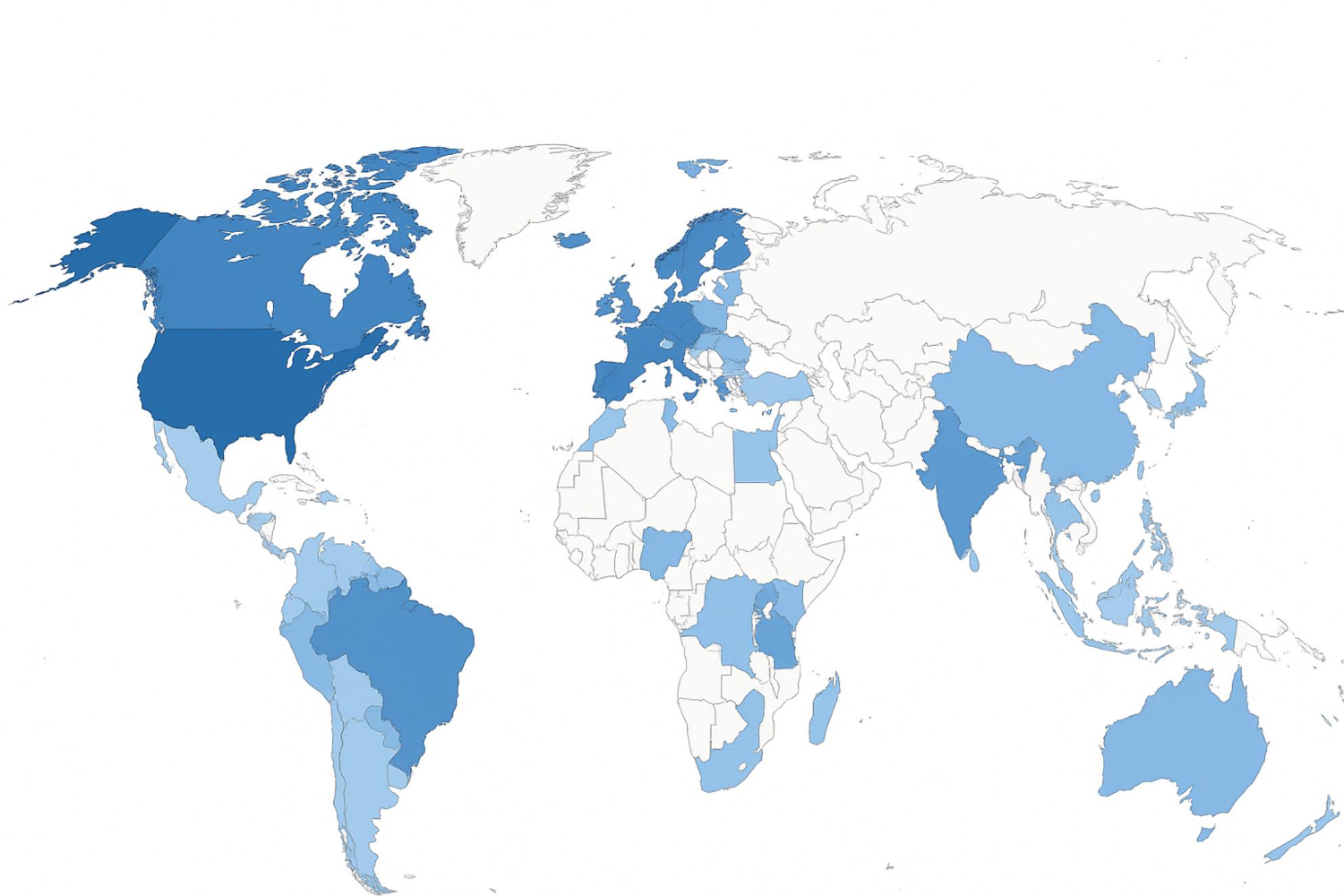
Global distribution of the studies on the impacts of climate change on mental health

A systematic search was conducted across Scopus, Web of Science, PubMed, and Google Scholar, with a cutoff date of October 23, 2024. The search initially identified 25,450 records: 11,616 from Scopus, 3,606 from PubMed, 10,068 from Web of Science, and 100 from Google Scholar. After removing duplicates, 11,889 unique records remained for screening. Titles and abstracts were assessed for relevance, resulting in 52 full-text articles evaluated for eligibility. Of these, 30 studies were excluded due to irrelevance, insufficient methodological rigor, or unavailability of full texts. Ultimately, 22 systematic reviews met the inclusion criteria and were included in the final synthesis (Fig. 1). The included studies span a broad geographical range, covering high-income countries (e.g., Canada, Greece, Australia), low- and middle-income settings (e.g., sub-Saharan Africa, South and Southeast Asia, Latin America), and global or multinational contexts. Temporally, most studies were published from 2022 onwards, indicating a growing research focus on climate change and mental health (Table 3; Fig. 2). Populations studied were diverse, including children, adolescents, older adults, women, indigenous groups, individuals with pre-existing health conditions, and occupationally exposed groups such as firefighters. Several studies also investigated general populations with specific subgroups (e.g., university students, environmental activists). This diversity highlights both the broad impact of climate-related exposures on mental health and the differential vulnerability across populations. 1

### Descriptive characteristics, Geographical and temporal distribution of studies

The studies covered a wide geographical range, including global research in regions like the United States, Kenya, and India, as well as specific high-income countries such as Canada, Greece, and Australia. Some focused on regional contexts like the Arctic and German-speaking Europe, while others explored low- and middle-income settings in sub-Saharan Africa, South and Southeast Asia, and Latin America. A few studies were multinational, involving both high- and low-income countries. Additionally, some research was conducted in single-country contexts, including Iran and the Philippines. Most studies were published in 2024 (*n* = 9), followed by three each in 2023 and 2022, three in 2021, two in 2020, and one each in 2018 and 2017 (Table 3). Figure 2 illustrates the geographic distribution of the studies included in the analysis, highlighting their prevalence across various countries and regions.

**Table 3:** Descriptive characteristics of the included articles

Title		First Author	Year	Country/Region	Study Population	Method
1	Intersections of Intimate Partner Violence and Natural Disasters: A Systematic Review of the Quantitative Evidence	Jennifer Boddy	2024	Global (USA, Kenya, Bangladesh, China, Nepal, Haiti, India, New Zealand, etc.)	Adult women (≥ 18), especially those exposed to natural disasters	Systematic review of quantitative evidence
2	Exposure to Wildfires and Mental Health Problems among Firefighters: A Systematic Review	Isabelle Bonita	2024	Israel, Canada, Greece, Australia	Firefighters (primarily wildland firefighters involved in wildfires)	Systematic review
3	Effect of Extreme Weather Events on Mental Health: A Narrative Synthesis and Meta-Analysis for the UK	Joana Cruz	2020	United Kingdom	General UK population exposed to floods, storms, heatwaves	Narrative synthesis and meta-analysis
4	Climate Change and Indigenous Mental Health in the Circumpolar North: A Systematic Review to Inform Clinical Practice	Laurence Lebel	2022	Circumpolar North (Canada, Alaska/USA, Greenland, Sápmi—Sweden/Norway/Finland, Arctic Russia)	Indigenous peoples (e.g., Inuit, Sámi, Dene, Cree, Gwich’in, Iñupiat, Aleut, Athabaskan, Yupik)	Systematic review
5	Adverse Health Effects of Climate Change and Air Pollution in People with Disabilities: A Systematic Review	Nakyung Rhim	2024	Global	People with disabilities	Systematic review
6	Weather and Suicide: Association Between Meteorological Variables and Suicidal Behavior—A Systematic Qualitative Review	C. Pervilhac	2020	German-speaking Europe (e.g., Germany, Austria, Switzerland)	General population	Systematic qualitative review
7	Impact of Extreme Weather Events on Sub-Saharan African Child and Adolescent Mental Health: The Implications of a Systematic Review of Sparse Research Findings	Hanna-Andrea Rother	2021	Sub-Saharan Africa (Namibia, Nigeria, Chad, etc.)	Children and adolescents	Systematic review
8	Understanding the Spectrum of Anxiety Responses to Climate Change: A Systematic Review of the Qualitative Literature	Catriona Soutar	2022	Multinational: mostly high-income/Western, plus South Korea, Ghana, Tuvalu, Fiji, Cyprus, New Zealand	General public	Systematic review of qualitative literature
9	A Systematic Review of Mental Health and Climate Change in the Philippines	Villarino Resti Tito	2024	Philippines	General population	Systematic review
10	Extreme Weather Events in Europe and Their Health Consequences—A Systematic Review	Veronika Weilnhammer	2021	Europe (e.g., Spain, UK, France, Italy, Hungary, Germany, etc.)	General public	Systematic review
11	Ambient Temperature, Sunlight Duration, and Suicide: A Systematic Review and Meta-Analysis	Jiaojiao Gao	2018	Multinational (Brazil, Australia, Kazakhstan, Korea, UK, Canada, Italy, Taiwan, Austria, New Zealand, Greece, Norway, Colombia, etc.)	General population	Systematic review and meta-analysis
12	Associations Between Ambient Temperature and Suicide: A Systematic Review	Andrej M. Grjibovski	2023	Global	General population	Systematic review
13	Recent Techniques in Determining the Effects of Climate Change on Depressive Patients: A Systematic Review	Nur Izzati Ab Kader	2022	Various countries	Patients with depressive disorder	Systematic review
14	Impact of Climate-Induced Floods and Typhoons on Geriatric Disabling Health Among Older Chinese and Filipinos: A Cross-Country Systematic Review	Joseph Kimuli Balikuddembe	2024	China and the Philippines	Older adults (≥ 60 years)	Systematic review
15	Climate Change Perception and Mental Health: Results from a National Survey	Vincenza Gianfredi	2024	Global	General population across all ages; some studies focus on youth, university students, adolescents, and subgroups (e.g., environmental activists, parents, urban/rural residents)	Systematic review of the literature
16	Climatic and Meteorological Exposure and Mental and Behavioral Health: A Systematic Review and Meta-Analysis	Dongying Li	2023	Global	General population exposed to climatic/meteorological variables	Systematic review and meta-analysis
17	Reporting Evidence on the Environmental and Health Impacts of Climate Change on Indigenous Peoples of Atlantic Canada: A Systematic Review	Pelin Kınay	2023	Canada	Indigenous communities in Atlantic Canada	Systematic review
18	Health Conditions in Land Transport Workers and Climate Change: Exploratory Systematic Review	Wilder Hernández	2024	Multinational (Latin America, USA, Switzerland, Peru; focus on Colombia/Latin America)	Land transport workers (drivers; public/private sectors; all ages; both sexes; varied socioeconomic backgrounds)	Exploratory systematic review (PRISMA-based)
19	Impact of Extreme Weather Events on Mental Health in South and Southeast Asia: Two Decades of Systematic Review of Observational Studies	Muhammad Mainuddin Patwary	2024	Bangladesh, India, Philippines, Pakistan, Nepal, Sri Lanka, Thailand, Vietnam, Indonesia, etc.	South and Southeast Asian populations	Systematic review
20	Is There an Association Between Hot Weather and Poor Mental Health Outcomes? A Systematic Review and Meta-Analysis	Jingwen Liu	2021	Global	General population	Systematic review and meta-analysis
21	The Incidence Rates of Suicide Attempts and Successful Suicides in Seven Climatic Conditions in Iran (2001–2014): A Systematic Review and Meta-Analysis	Salman Deliri	2017	Iran	General Iranian population	Systematic review and meta-analysis
22	Health Risks of Climate Change in Australia: An Umbrella Review	Michael Tong	2024	Australia	General Australian population; especially those with chronic conditions/disability, socioeconomically disadvantaged groups, and people exposed to wildfires, floods, droughts, heatwaves	Umbrella review

### Study populations

The studies examined diverse populations affected by climate change, including women aged 18 or older at risk of natural disasters; firefighters involved in wildland fires; the general population in the UK facing extreme weather events; and indigenous communities in polar regions, such as the Inuit, Sami, and Cree. Other groups included individuals with disabilities, children, patients with depression, and older adults. Some studies analyzed general populations, including subgroups such as youth, university students, and environmental activists. Additional populations included communities in Atlantic Canada, individuals in the Philippines affected by climate disasters, transport workers, and groups in South and Southeast Asia, as well as those in Iran and Australia affected by severe events such as wildfires and floods (Table 4). Comparative analysis based on the included systematic reviews indicates that children, older adults, and women consistently exhibit higher vulnerability across both acute (e.g., storms, floods) and chronic (e.g., prolonged heat, drought) climate exposures, whereas occupational groups such as firefighters are particularly susceptible to environmental hazards specific to their work context.

### Contextual and risk factors

Analysis of the included high-quality systematic reviews consistently indicated that climate-related mental health outcomes disproportionately affect vulnerable populations, including children, adolescents, older adults, women, ethnic minorities, indigenous populations, and individuals with pre-existing health conditions. Socioeconomic disadvantages—such as low income, limited education, unemployment, insecure housing, and lack of insurance—emerged as recurrent determinants of susceptibility. Exposure to acute climate events (including wildfires, floods, storms, heatwaves, and droughts) and residence in disaster-prone areas further modulated risk. Individual factors, such as prior trauma, substance use, disability, and limited adaptive capacity, influenced vulnerability, whereas community-level resources, including social support and resilience, appeared to mitigate adverse outcomes. Geographic and climatic contexts (urban vs. rural, regional temperature extremes) interacted with these determinants, amplifying risk in specific populations. These findings underscore consistent patterns across multiple reviews and highlight the critical role of socio-environmental and contextual determinants in shaping psychiatric vulnerability (Table 4). Analysis of contextual and risk factors suggests a hierarchical pattern: socioeconomic disadvantages (e.g., low income, limited education, insecure housing) consistently emerged as primary determinants of mental health vulnerability, whereas individual-level factors (e.g., prior trauma, disability, limited adaptive capacity) appeared more context-dependent and varied across populations and exposure types. Intersecting vulnerabilities related to gender, socioeconomic status, and geographic location further amplify psychiatric risk, with women in low-income rural areas experiencing particularly compounded exposure to climate-related mental health outcomes.

### Mechanisms of impact: acute vs. chronic events

Evidence from the systematic reviews suggests that climate change influences mental health through distinct but interacting pathways. Evidence for acute pathways is strongest among children, older adults, and pregnant women directly exposed to extreme weather events, while chronic pathway evidence predominantly derives from low-income, rural, and agriculture-dependent populations experiencing sustained environmental stressors, including prolonged droughts, rising temperatures, and ecosystem degradation. Acute events (such as storms, floods, wildfires, and heatwaves) were generally associated with immediate psychological distress, PTSD, anxiety, and depression, particularly among highly exposed groups, including children, older adults, pregnant women, and individuals with chronic illness. Chronic climate changes—including sustained temperature increases, prolonged droughts, and ecosystem degradation—were linked to gradual elevations in depression, anxiety, stress-related disorders, and substance use, often mediated by economic stress, food insecurity, and social inequalities. Acute and chronic pathways frequently co-occurred, potentially amplifying psychiatric risk. These mechanistic insights suggest that effective interventions may need to simultaneously address immediate and long-term mental health consequences (Table 4).

### Psychiatric outcomes

Across reviews, PTSD, anxiety, depression, and stress-related disorders demonstrated the most consistent associations with climate-related exposures. Evidence for substance use disorders, mood disorders, suicidal ideation, and sleep disturbances was observed in multiple reviews but with some heterogeneity in populations and outcomes. Findings for schizophrenia, bipolar disorder, cognitive impairments, and personality disorders were relatively limited, often reported in isolated studies or context-specific settings. Developmental and behavioral disorders in children were predominantly observed in contexts of repeated or severe climate exposure. These patterns identify priority areas for mental health monitoring and intervention while highlighting gaps in longitudinal data, treatment response, and culturally specific outcomes (Table 4).

### Impact on social and occupational wellbeing

Systematic reviews consistently reported that climate-related events were associated with disruptions in social cohesion, occupational functioning, and community stability. Increased risks of displacement, violence, loss of homes and livelihoods, occupational burnout, and maladaptive coping strategies (including substance use) were particularly noted among marginalized and low-resource populations. Extreme temperatures were linked to higher hospital admissions, self-harm, and suicide, particularly in tropical and low-income regions. These findings reinforce the necessity of resilience-focused public health strategies and identify subpopulations where targeted interventions may yield the greatest benefit. Intersecting vulnerabilities related to gender, socioeconomic status, and geographic location amplify the mental health impacts of climate events, with women living in low-income rural areas experiencing particularly compounded risk (Table 4).

**Table 4:** Domains and metrics utilized for evaluating the effects of climate change on mental health in the articles reviewed (*n* = 22)

Outcome/Exposure	Population/Vulnerable groups	Evidence quality (JBI)	Acute vs. chronic	Mechanistic pathway/analytical insight	Referenced
Contextual & socioeconomic risk factors (low income, limited education, unemployment, insecure housing, lack of insurance)	Children, Adolescents, Older adults, Women, Ethnic/minority communities, Indigenous populations, Individuals with pre-existing conditions	High	Chronic	Socioeconomic disadvantage modulates vulnerability; individual factors (trauma, disability) interact with community resources (social support, resilience) to shape psychiatric outcomes	[1–22], All articles except [4, 21]
Acute climate events (wildfires, floods, storms, heatwaves, droughts)	Children, Adolescents, Older adults, Pregnant women, Indigenous populations, Individuals with pre-existing conditions	High	Acute	Immediate environmental and occupational exposures elicit psychological distress; acute physical hazards interact with pre-existing vulnerabilities	[27, 29, 36, 38–46]
Chronic climate changes (rising temperatures, prolonged droughts, altered precipitation, sea level rise, permafrost thaw, ecosystem degradation)	Agriculture-dependent, Rural, Low-income populations	High	Chronic	Long-term environmental stressors exacerbate socioeconomic and food security pressures; cumulative exposures amplify vulnerability and mental health risk	[27, 28, 32–34, 36, 39–47]
PTSD	Children, Adolescents, Adults, Older adults, Indigenous populations, Exposed occupational groups	High	Acute	Acute trauma exposure combined with environmental stressors contributes to persistent psychological distress; contextual factors mediate severity	[29, 30, 32, 36–39, 41, 43, 44]
Anxiety	All populations	High	Acute & Chronic	Psychological stress arises from both acute and chronic climate-related exposures; socioeconomic and environmental factors modulate response	[3, 27–40, 42–47]
Depression	Children, Adolescents, Adults, Older adults	High	Acute & Chronic	Chronic environmental and socioeconomic stressors interact with individual susceptibility to influence depressive symptoms	[3, 28–30, 33, 35–41, 43, 46, 47]
Suicidal ideation/self-harm	Children, Adolescents, Adults, Older adults	High	Acute & Chronic	Psychological distress and social disruption amplify risk; acute shocks may precipitate immediate crisis, while chronic stressors exacerbate cumulative vulnerability	[3, 28–30, 33, 35–41, 43, 46, 47]
Substance use disorders	Adolescents, Adults, Exposed occupational groups	Moderate	Acute & Chronic	Maladaptive coping emerges in response to combined environmental, socioeconomic, and occupational stressors; patterns vary by context	[3, 28–30, 33, 35–41, 43, 46, 47]
Burnout/Occupational stress	Adults, Exposed occupational groups	Moderate	Acute	High workloads and environmental hazards interact to produce work-related stress; acute event exposure precipitates burnout	[38]
Sleep disorders	Children, Adolescents, Adults	Moderate	Acute & Chronic	Environmental disruption, psychological stress, and individual vulnerability converge to impair sleep patterns	[28, 29, 34, 35, 39, 44–46]
Mood disorders/Bipolar/Schizophrenia	Adults	Low	Chronic	Individual predisposition interacts with chronic socioeconomic and environmental stressors; evidence limited and context-dependent	[30, 33, 35, 39, 41, 44, 45]
Cognitive impairments	Children, Adults	Low	Chronic	Developmental disruption and environmental exposures may impair cognition; limited evidence	[3, 31]
Personality disorders	Adults	Low	Chronic	Social disruption and individual vulnerability influence outcomes; evidence is preliminary	[33, 45]
Developmental & behavioral disorders in children	Children	Moderate	Chronic	Repeated/severe environmental exposures interact with social stressors to affect development	[29, 30, 45]
Social & occupational wellbeing (violence, displacement, loss of homes, job loss, migration, social isolation)	Children, Adolescents, Older adults, Women, Low-income households, Indigenous populations, Exposed occupational groups	High	Acute & Chronic	Environmental and socioeconomic stressors destabilize social cohesion and occupational functioning; vulnerable groups experience compounded effects	[27–47]
Extreme temperatures (hospital admissions, mortality, self-harm, suicide)	Tropical, Low-income, Infrastructure-poor populations	High	Acute & Chronic	Acute and chronic thermal stress interacts with socioeconomic disadvantage and pre-existing vulnerability to increase health risk	[27–47]
Gender-based risks/economic instability	Women, Low-income households	Moderate	Chronic	Socioeconomic and gendered vulnerabilities interact with chronic environmental stressors; dependence on social support mediates outcomes	[30, 34, 37, 39, 42, 45]

## Discussion

**Fig. 3:**
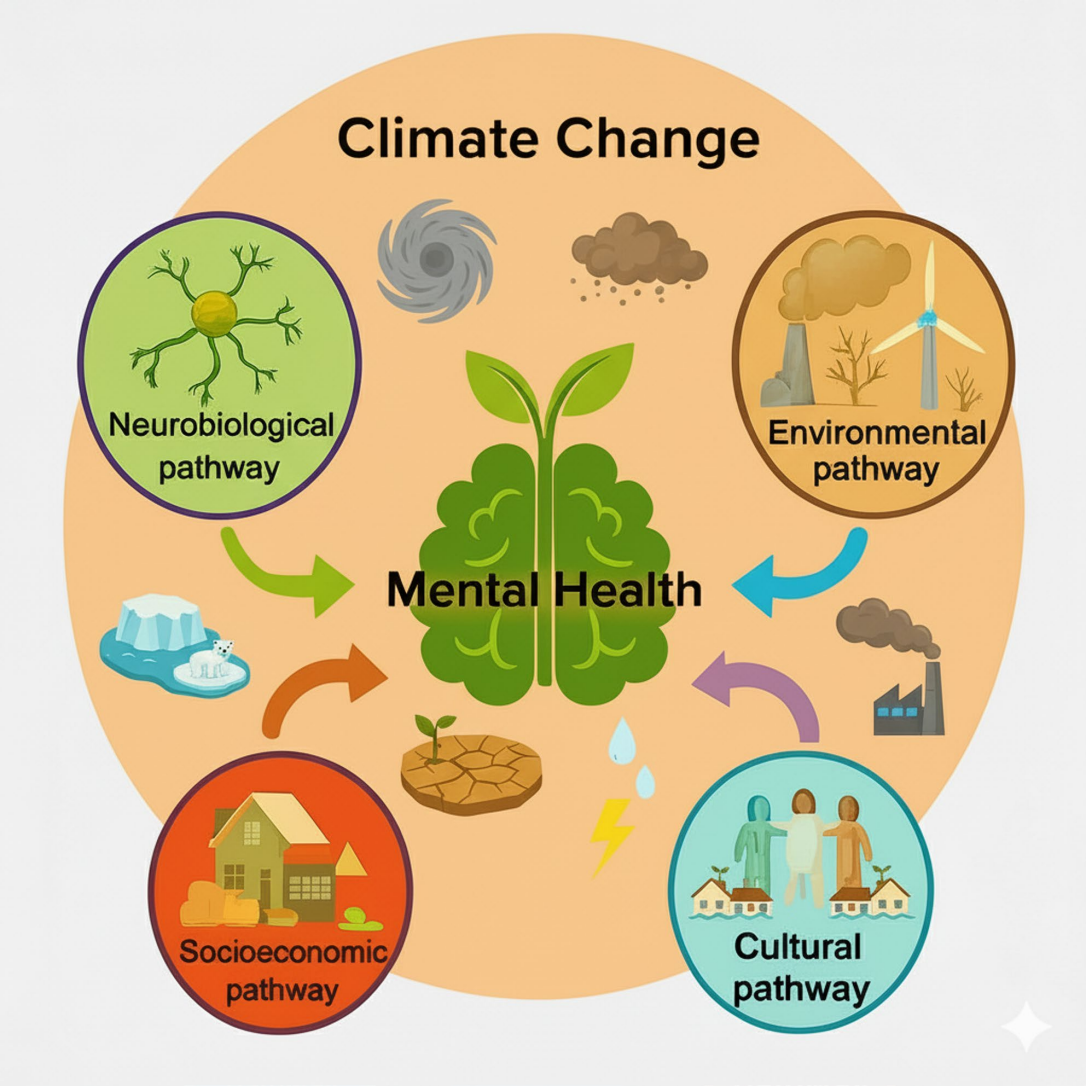
Pathways Through which climate change impacts mental health

From a psychiatric perspective, the mental health consequences of climate change extend beyond transient distress and may manifest as clinically significant disorders. Evidence directly extracted from the included systematic reviews is distinguished from supporting theoretical or mechanistic explanations drawn from complementary literature, allowing readers to differentiate empirical findings from conceptual elaboration. Documented increases in anxiety, depressive symptoms, and trauma-related reactions correspond to established diagnostic categories, including post-traumatic stress disorder (PTSD), major depressive disorder, generalized anxiety disorder, and adjustment disorders. Framing climate-related psychological responses within psychiatric nosology enhances clinical interpretability and underscores the relevance of these findings for targeted mental health assessment and intervention. Evidence indicates that climate-related stressors exert both direct and indirect effects on mental health. Direct consequences include anxiety, depression, PTSD, and suicidal ideation, whereas indirect effects arise from disruptions in livelihoods, forced migration, poverty, limited access to essential resources, and weakened social cohesion [39, 45]. This study adopts a four-pathway conceptual framework designed to organize heterogeneous findings into a coherent explanatory structure. The neurobiological pathway encompasses stress physiology, neuroendocrine responses, and biological mechanisms associated with acute and chronic environmental stress. The socioeconomic pathway captures the mental health effects of economic instability, displacement, livelihood disruption, and structural inequalities. The environmental pathway reflects the direct and indirect psychological consequences of climate-related exposures and ecological changes. The cultural pathway addresses processes involving identity disruption, solastalgia, community cohesion, and culturally mediated resilience. These pathways are conceptualized as dynamically interacting rather than discrete domains, collectively shaping patterns of psychiatric vulnerability, risk distribution, and adaptive responses. The structure of the proposed framework, including its primary domains, representative mechanisms, and associated psychiatric outcomes, is summarized in Table 5. Vulnerable populations—including children, adolescents, older adults, women, ethnic and minority communities, and individuals with pre-existing physical or mental health conditions—are disproportionately affected, highlighting the importance of considering population-specific susceptibilities in psychiatric evaluation. Based on the findings of this study, a four-pathway conceptual framework is proposed to explain the relationship between climate change exposures and mental health outcomes (Fig. 3). Women, particularly in low-income or rural settings, experience compounded vulnerability due to care responsibilities, greater exposure to resource insecurity, and limited access to mental health services. Gender intersects with socioeconomic and geographic factors, amplifying mental health risks during climate-related events. The framework encompasses neurobiological, socioeconomic, environmental, and cultural pathways, which collectively capture how acute and chronic climate stressors interact with biological, social, and environmental systems to influence psychiatric vulnerability. Cultural and community factors further modulate psychological responses, shaping resilience or susceptibility. This integrative model provides a coherent explanation of observed psychiatric outcomes and identifies high-risk populations, while the subsequent sections provide a detailed examination of each pathway and its implications for mental health.

**Table 5:** Conceptual four-pathway framework associated climate-related exposures to psychiatric outcomes

Pathway	Conceptual domain	Representative mechanisms	Illustrative psychiatric outcomes	Vulnerability modifiers
Neurobiological pathway	Biological and physiological stress responses	Stress physiology, neuroendocrine dysregulation, allostatic load, sleep disruption, inflammatory processes	PTSD, anxiety disorders, depressive symptoms, sleep disturbances	Pre-existing mental disorders, age, chronic illness, genetic susceptibility
Socioeconomic pathway	Structural and economic determinants of mental health	Economic instability, livelihood disruption, displacement, housing insecurity, resource scarcity	Depression, anxiety, substance use, suicidal ideation, stress-related disorders	Income level, employment status, social inequality, access to healthcare
Environmental pathway	Direct and indirect exposure to climate-related events	Exposure to extreme events, heat stress, ecological degradation, environmental uncertainty	PTSD, anxiety, psychological distress, mood disturbances	Exposure severity, duration, frequency, geographic context
Cultural pathway	Psychosocial and identity-related processes	Identity disruption, solastalgia, community fragmentation, culturally mediated coping/resilience	Anxiety, depression, grief-related responses, stress-related disorders	Cultural attachment, social cohesion, community resilience

***All outcomes are presented based on high-quality systematic reviews and categorized according to the Joanna Briggs Institute (JBI) appraisal. Acute vs. chronic classification reflects the temporal nature of climate-related exposures. Mechanistic pathways***

1. **Neurobiological pathways**:

Included reviews suggested that climate change may affect mental well-being through several neurobiological pathways. Severe events such as storms, floods, and heatwaves were associated with activation of the hypothalamic-pituitary-adrenal (HPA) axis and sympathetic nervous system, potentially increasing cortisol and adrenaline levels. Prolonged stress exposure may contribute to hippocampal dysfunction and heightened amygdala reactivity, which could increase anxiety and emotional sensitivity [33, 41, 43, 47]. Prolonged activation of the HPA axis contributes to anxiety and depression, particularly among children and older adults, consistent with findings across the included systematic reviews. These stress-related neurobiological responses may be moderated by gender-related factors, including differential caregiving burdens, exposure patterns, and hormonal influences, potentially increasing vulnerability among women in some contexts. This highlights the clinical relevance of neurobiological pathways without overextending into detailed mechanistic description. The hippocampus plays a vital role in memory encoding and consolidation, and its impairment from climate-related trauma can lead to intrusive thoughts, flashbacks, and nightmares, hindering emotional recovery. Chronic stress activates the amygdala, causing hyperarousal, anxiety, and emotional dysregulation, while reduced control from the prefrontal cortex results in emotional volatility. In older adults, chronic stress can worsen depressive symptoms and accelerate cognitive decline. It suggests that climate change impacts mental health through neurobiological pathways, with prolonged exposure leading to changes in brain structure and function, increasing the risk of disorders like depression and PTSD, especially in developing children and adolescent [29, 44]. Evidence consistently demonstrates associations with anxiety, depression, and PTSD across multiple populations and reviews, whereas findings for substance use disorders, cognitive impairments, and personality disorders are more heterogeneous and context-specific. To counteract these neurobiological effects, mental adaptation activities and mindfulness-based interventions—such as yoga, breathwork, and meditation—have been shown to enhance prefrontal cortex functionality while concurrently reducing amygdala hyperactivity. It promotes improved emotional regulation and resilience [48, 49]. Mindfulness and physical activity can help restore balance in the HPA axis. These strategies lower stress hormone levels and improve emotional regulation in response to climate-related psychological challenges [50]. Numerous studies have shown that mindfulness-based interventions can modulate HPA axis activity and reduce cortisol secretion. These findings indicate that mindfulness may enhance the neurobiological regulation of the stress response, ultimately lower stress-induced physiological arousal and improving mental health outcomes [50, 51].

2. **Socioeconomic pathway**:

Socioeconomic factors critically shape the mental health impacts of climate change. Food insecurity, income volatility, and economic uncertainty generate persistent stress, contributing to anxiety, depression, and PTSD, particularly among vulnerable populations such as agricultural workers, children, and older adults [29, 30, 33, 43]. These burdens may disproportionately affect women, particularly those with unpaid caregiving responsibilities, informal employment, or limited financial autonomy. Climate-related disasters—floods, droughts, and livelihood disruptions—exacerbate economic strain and disproportionately affect those reliant on ecosystem services [52, 53]. Social capital and robust community networks mitigate these effects [54]. Economic instability, including job loss and sector-specific stress in agriculture or emergency services, further amplifies psychological distress and vulnerability to chronic anxiety and depression [55–57]. The evidence for anxiety, depression, and PTSD related to socioeconomic stressors is robust and observed across diverse populations, while associations with other psychiatric outcomes such as substance use disorders or behavioral disorders vary according to context and study design. Disruptions in social contexts—such as breakdowns in support networks or forced relocation—intensify trauma and feelings of helplessness, particularly in children [2, 3, 58, 59]. Incorporating psychosocial support, strengthening community networks, and promoting resilience-based mental health education are essential strategies to reduce the psychological consequences of climate-related socioeconomic disruptions [60].

3. **Environmental pathway**:

Environmental factors, including air pollution and extreme temperatures, contribute to cognitive deficits and psychological stress, particularly in urban areas with limited infrastructure. Vulnerable populations—children, older adults, and individuals with pre-existing health conditions—experience higher risks of anxiety, depression, PTSD, social disconnection, and cognitiv dysfunction. Gendered occupational roles, household responsibilities, and differential access to cooling, transport, or healthcare may further shape exposure and recovery [61]. Exposure to poor air quality, thermal stress, and environmental pollution has been associated with worsened depressive symptoms, heightened physiological stress, and diminished psychological resilience, with effects more pronounced in communities near industrial zones or with limited resources [2, 19, 29, 30, 33, 43, 44, 58, 59, 62–66]. Consistent evidence supports impacts on anxiety, depression, and PTSD in high-exposure populations, whereas findings for cognitive impairments, sleep disorders, and mood disorders are more variable and context-dependent. Focusing on the environmental pathway is essential for mitigating the mental health impacts of climate-related hazards. Strategies such as improving air quality, creating urban green spaces, reducing heat stress through urban design, ensuring stable access to food, and controlling industrial pollution can alleviate psychological distress and enhance resilience against cognitive and psychiatric consequences [67, 68].

4. **Cultural pathway**:

Climate change affects mental health through cultural pathways, as values, beliefs, and traditional practices shape how communities cope with environmental stressors and build resilience. Collectivist cultures may buffer psychological stress, whereas individualistic settings can exacerbate it [28, 30, 40, 44]. Gender norms within communities may also influence help-seeking behaviors, emotional expression, caregiving expectations, and access to mental health support.Indigenous populations are particularly vulnerable due to the loss of ancestral lands, cultural heritage, and traditional livelihoods, leading to environmental grief and heightened risk of anxiety, depression, and PTSD [28–30, 32, 37, 40, 69, 70]. While anxiety, depression, and PTSD are consistently reported across cultural and community contexts, evidence regarding the effects on other psychiatric outcomes is limited or context-specific, reflecting heterogeneity in cultural resilience and exposure. Historical marginalization and limited access to culturally competent mental health services further compound these risks. Community-based interventions that integrate culturally relevant recovery programs, preserve heritage, and strengthen communal rituals can mitigate psychological distress and enhance resilience [71–73]. Educational initiatives that reinforce culturally and environmentally oriented values can support social cohesion and reduce isolation. However, factors such as migration, globalization, and insufficient access to quality education constrain participation and resilience, highlighting persistent structural barriers [74, 75]. The findings of this systematic review are inherently limited by the quality, heterogeneity, and scope of the included systematic reviews. Differences in study populations, measurement tools, and contextual factors may influence generalizability and should be considered when interpreting the results. Overall, climate change exerts multidimensional impacts on mental health through neurobiological, socioeconomic, environmental, and cultural pathways. Vulnerable populations, particularly women, children, older adults, and marginalized communities, face amplified risk. These findings underscore the need for targeted mental health interventions, culturally competent care, and policies addressing structural inequities, thereby integrating psychiatric considerations into climate adaptation strategies. Future research should employ quantitative, qualitative, and longitudinal approaches to evaluate the impact of cultural practices and support networks on mental health outcomes in climate-affected populations [76].

### Limitations and future research directions

This review provides important insights into the psychological consequences of climate change; however, several limitations should be acknowledged. A substantial proportion of the available literature is derived from high-income countries, resulting in the underrepresentation of low-income nations and certain demographic groups. This imbalance restricts the generalizability of current findings and highlights the need for more geographically and socioeconomically diverse research. Furthermore, the heterogeneity of study designs and methodologies—encompassing both qualitative and quantitative approaches—has contributed to variability in data quality and outcome interpretation. Standardization of research methodologies, including the use of comparable measurement tools, study designs, and outcome variables, is therefore essential to enhance cross-study comparability and strengthen future evidence synthesis. Although numerous studies report associations between climate-related events and mental health outcomes such as anxiety, depression, and post-traumatic stress disorder, establishing causal relationships remains challenging. The complex interplay of environmental, social, and economic factors necessitates the use of longitudinal and mechanistic research designs to clarify causal pathways. Future investigations should extend beyond short-term psychological effects to examine long-term impacts, including chronic stress, developmental vulnerability, and potential intergenerational consequences. Greater attention should also be directed toward understanding the biological mechanisms underlying climate-related psychological responses, as well as the cultural and social dimensions that shape mental health outcomes across different populations. Addressing these gaps carries significant clinical and public health implications. Early identification of individuals exposed to climate-related stressors may facilitate targeted screening for mood, anxiety, and trauma-related disorders. Improved understanding of maladaptive stress responses and resilience mechanisms may inform the development of preventive psychosocial and pharmacological interventions. At the systems level, the findings underscore the necessity of strengthening mental health service preparedness, particularly in disaster-prone and socioeconomically vulnerable regions. Enhancing workforce training, expanding community-based psychiatric services, and developing equity-focused adaptation strategies will be critical for mitigating the long-term psychiatric burden associated with climate changeThese findings have meaningful implications for psychiatric prevention and early intervention. Identification of individuals exposed to climate-related stressors may facilitate targeted screening for PTSD, mood disorders, and anxiety disorders. Early recognition of maladaptive stress responses, emotional dysregulation, and chronic stress patterns may allow clinicians to implement preventive psychosocial and pharmacological interventions. The identified pathways may also represent potential therapeutic targets, particularly for resilience-enhancing and stress-regulation strategies. At the mental health systems level, the results underscore the need for strengthening psychiatric service preparedness. Climate-related events may increase demand for psychiatric care, particularly in disaster-prone and socioeconomically vulnerable regions. Workforce training in trauma-informed care, crisis intervention, and climate-related psychological stressors may be necessary. Community-based psychiatric services, integrated care models, and preventive mental health programs may play a critical role in mitigating long-term psychiatric morbidity associated with climate change.

## Conclusion

Climate change should be recognized not only as an environmental crisis but also as an emerging determinant of psychiatric disorders. Evidence indicates that climate-related exposures impact mental health through four interrelated pathways—neurobiological, socioeconomic, environmental, and cultural—which collectively shape psychiatric vulnerability. Gender should be understood not only as a marker of vulnerability, but as a cross-cutting determinant shaping exposure, coping capacity, caregiving burden, access to care, and long-term mental health outcomes. Neurobiological pathways involve stress-mediated alterations in brain function and hormonal regulation, increasing susceptibility to anxiety, depression, and PTSD. Socioeconomic pathways, including financial hardship, displacement, and limited access to resources, exacerbate psychological distress. Environmental pathways, such as exposure to pollutants, extreme temperatures, and ecosystem disruption, contribute to cognitive decline and emotional dysregulation. Cultural pathways, including erosion of identity, disruption of traditional practices, and weakened community cohesion, amplify mental health risks, particularly among Indigenous and marginalized populations. These insights underscore the need to integrate psychiatric prevention such as cognitive behavioral therapy, trauma focused therapies, screening and early detection, and culturally competent interventions into climate adaptation policies. Strengthening mental health services, enhancing workforce preparedness, expanding community-based care, and prioritizing vulnerable populations are essential strategies to mitigate the psychological consequences of climate change and enhance resilience across affected communities.

## Data Availability

The datasets used and/or analyzed during the current study are available from the corresponding author on reasonable request.

## Footnotes

1 Figure 1: PRISMA flow diagram illustrating records identified, screened, excluded, and included. Rationale: All key counts (by database, screening, exclusions) are included in the main text to maintain transparency and clarity without requiring supplementary material. Analytic insight: The upward trend in publications from 2022 onwards reflects increasing scientific interest in the psychiatric consequences of climate change.

## References

[1] Jung Y-J, Khant N, Kim H, Namkoong S. Impact of Climate Change on Waterborne Diseases: Directions towards Sustainability. Water. 2023;15:1298.

[2] Mousavi A, Ardalan A, Takian A, Ostadtaghizadeh A, Naddafi K, Bavani AM. Climate change and health in Iran: a narrative review. J Environ Health Sci Eng. 2020;18(1):367–78. 32399247 10.1007/s40201-020-00462-3 PMC7203306

[3] Gianfredi V, Mazziotta F, Clerici G, Astorri E, Oliani F, Cappellina M, et al. Climate change perception and mental health. Results from a systematic review of the literature. Eur J Investig Health Psychol Educ. 2024;14(1):215–29. 38248134 10.3390/ejihpe14010014 PMC10814599

[4] Goniewicz K, Burkle FM, Khorram-Manesh A. Transforming global public health: climate collaboration, political challenges, and systemic change. J Infect Public Health. 2025;18(1):102615. 39644717 10.1016/j.jiph.2024.102615

[5] Chukwuma Sr C. Invariance of Extreme Hydrologic Events and Climate Change in the Risk Reduction on Environment and Health. Grn Int J Apl Med Sci. 2025;3(2):92–102.

[6] Mani ZA, Khorram-Manesh A, Goniewicz K. Global health emergencies of extreme drought events: historical impacts and future preparedness. Atmosphere Basel. 2024;15(9):1137.

[7] Léger-Goodes T, Malboeuf-Hurtubise C, Mastine T, Généreux M, Paradis P-O, Camden C. Eco-anxiety in children: a scoping review of the mental health impacts of the awareness of climate change. Front Psychol. 2022;13:872544. 35959069 10.3389/fpsyg.2022.872544 PMC9359205

[8] Barati AA, Azadi H, Movahhed Moghaddam S, Scheffran J, Dehghani Pour M. Agricultural expansion and its impacts on climate change: evidence from Iran. Environ Dev Sustain. 2024;26(2):5089–115.

[9] Salehi S, Ardalan A, Garmaroudi G, Ostadtaghizadeh A, Rahimiforoushani A, Zareiyan A. Climate change adaptation: a systematic review on domains and indicators. Nat Hazards. 2019;96(1):521–50.

[10] Abbasi H. The effect of climate change on depression in urban areas of Western Iran. BMC Res Notes. 2021;14(1):155. 33892805 10.1186/s13104-021-05565-0 PMC8063425

[11] Aylward B, Cunsolo A, Vriezen R, Harper SL. Climate change is impacting mental health in North America: a systematic scoping review of the hazards, exposures, vulnerabilities, risks and responses. International Review of Psychiatry. 2022;34(1):34–50. 35584021 10.1080/09540261.2022.2029368

[12] Salehi S, Ardalan A, Ostadtaghizadeh A, Garmaroudi G, Zareiyan A, Rahimiforoushani A. Conceptual definition and framework of climate change and dust storm adaptation: a qualitative study. J Environ Health Sci Eng. 2019;17(2):797–810. 32030153 10.1007/s40201-019-00396-5 PMC6985416

[13] Lebel L, Paquin V, Kenny T-A, Fletcher C, Nadeau L, Chachamovich E, et al. Climate change and Indigenous mental health in the Circumpolar North: a systematic review to inform clinical practice. Transcultural Psychiatry. 2022;59(3):312–36. 34989262 10.1177/13634615211066698 PMC9160950

[14] Abdoli A, Olfatifar M, Eslahi AV, Moghadamizad Z, Samimi R, Habibi MA, et al. A systematic review and meta-analysis of protozoan parasite infections among patients with mental health disorders: an overlooked phenomenon. Gut Pathogens. 2024;16(1):7. 38282036 10.1186/s13099-024-00602-2 PMC10822187

[15] Pardon M, Dimmock J, Chande R, Kondracki A, Reddick B, Davis A, et al. Mental health impacts of climate change and extreme weather events on mothers. European journal of psychotraumatology. 2024;15(1):2296818. 38224060 10.1080/20008066.2023.2296818 PMC10791077

[16] Kirkbride JB, Anglin DM, Colman I, Dykxhoorn J, Jones PB, Patalay P, et al. The social determinants of mental health and disorder: evidence, prevention and recommendations. World psychiatry. 2024;23(1):58–90. 38214615 10.1002/wps.21160 PMC10786006

[17] Cosh SM, Ryan R, Fallander K, Robinson K, Tognela J, Tully PJ, et al. The relationship between climate change and mental health: a systematic review of the association between eco-anxiety, psychological distress, and symptoms of major affective disorders. BMC Psychiatry. 2024;24(1):833. 39567913 10.1186/s12888-024-06274-1 PMC11577747

[18] Ogbanga MM. Climate Change and Mental Health: The Unseen Consequences of a Changing Environment. 2024.

[19] Barbani SM. The indirect consequences of climate change and extreme environmental events on mental health: a literature review. J Public Health. 2025:1–14.

[20] Khazaee-Pool M, Pashaei T, Yazdani F, Ponnet K. Exploring healthcare workers’ perceptions and experiences regarding post-traumatic stress disorder after 2 years of the last global pandemic. BMC Health Serv Res. 2025;25(1):861. 40598425 10.1186/s12913-025-13004-0 PMC12219579

[21] Parsons ES, Jowell A, Veidis E, Barry M, Israni ST. Climate change and inequality. Pediatr Res. 2024:1–8. 10.1038/s41390-024-03443-6 PMC11772218 39075170

[22] Subroto S, Datta R. Perspectives of racialized immigrant communities on adaptability to climate disasters following the UN Roadmap for Sustainable Development Goals (SDGs) 2030. Sustain Dev. 2024;32(2):1386–400.

[23] Tannenbaum-Baruchi C, Ashkenazi I, Rapaport C. Risk inclusion of vulnerable people during a climate-related disaster: a case study of people with hearing loss facing wildfires. Int J Disaster Risk Reduct. 2024;103:104335.

[24] Kimutai J, Vautard R, Zachariah M, Tolasz R, Šustková V, Cassou C et al. Climate change and high exposure increased costs and disruption to lives and livelihoods from flooding associated with exceptionally heavy rainfall in Central Europe. 2024.

[25] Pasupuleti R, Orekanti ER. Resilience nexus with climate change, food security, mental health, and social stability in a changing world. In: Impact of Climate Change on Mental Health and Well-Being. IGI Global Scientific Publishing; 2024. p. 67–81.

[26] Ek C, Hébert JR, Friedman DB, Porter DE. Climate change, racism, and food insecurity: cyclical impacts of stressors exacerbate health disparities. J Racial Ethn Health Disparities. 2024;12:1. 39412743 10.1007/s40615-024-02202-x PMC12644168

[27] Weilnhammer V, Schmid J, Mittermeier I, Schreiber F, Jiang L, Pastuhovic V, et al. Extreme weather events in europe and their health consequences - A systematic review. Int J Hyg Environ Health. 2021;233:113688. 33530011 10.1016/j.ijheh.2021.113688

[28] Kinay P, Wang X, Augustine P, Augustine M. Reporting evidence on the environmental and health impacts of climate change on Indigenous Peoples of Atlantic Canada: a systematic review. Environ Res Clim. 2023. 10.1088/2752-5295/accb01.

[29] Patwary MM, Bardhan M, Haque MA, Moniruzzaman S, Gustavsson J, Khan MMH, et al. Impact of extreme weather events on mental health in South and Southeast Asia: A two decades of systematic review of observational studies. Environ Res. 2024;250:118436. 38354890 10.1016/j.envres.2024.118436

[30] Rother H-A, Hayward RA, Paulson JA, Etzel RA, Shelton M, Theron LC. Impact of extreme weather events on Sub-Saharan African child and adolescent mental health: the implications of a systematic review of sparse research findings✰. J Clim Change Health. 2022;5:100087.

[31] Soutar C, Wand APF. Understanding the spectrum of anxiety responses to climate change: a systematic review of the qualitative literature. Int J Environ Res Public Health. 2022. 10.3390/ijerph19020990. 35055813 10.3390/ijerph19020990 PMC8776219

[32] Rhim N, Lee S, Choi KH. Adverse health effects of climate change and air pollution in people with disabilities: a systematic review. Epidemiol Health. 2024;46:e2024080. 39363605 10.4178/epih.e2024080 PMC11832245

[33] Grjibovski A, Kobelev I, Kukalevskaya N, Popova Y, Baranov A. Associations between ambient temperature and suicide: а systematic review. Ekologiya cheloveka (Human Ecology). 2023. 10.17816/humeco569176.

[34] Ab Kader NI, Yusof UK, Khalid MNA, Nik Husain NR. Recent techniques in determining the effects of climate change on depressive patients: a systematic review. J Environ Public Health. 2022;2022:1803401. 35978588 10.1155/2022/1803401 PMC9377838

[35] Liu J, Varghese BM, Hansen A, Xiang J, Zhang Y, Dear K, et al. Is there an association between hot weather and poor mental health outcomes? A systematic review and meta-analysis. Environ Int. 2021;153:106533. 33799230 10.1016/j.envint.2021.106533

[36] Tong M, Okokon E, Vardoulakis S. Health risks of climate change in Australia: an umbrella review. J Clim Change Health. 2024;20:100347. 10.1016/j.joclim.2024.100347 PMC12851267 41647118

[37] Boddy J, Harris C, O’Leary P, Hohenhaus M, Bond C, Panagiotaros C, et al. Intersections of intimate partner violence and natural disasters: a systematic review of the quantitative evidence. Trauma Violence Abuse. 2024;25(4):3131–48. 38770897 10.1177/15248380241249145 PMC11370170

[38] Bonita I, Halabicky O, Liu J. Exposure to wildfires exposures and mental health problems among firefighters: a systematic review. Atmosphere. 2024;15:78.

[39] Cruz J, White PCL, Bell A, Coventry PA. Effect of extreme weather events on mental health: a narrative synthesis and meta-analysis for the UK. Int J Environ Res Public Health. 2020. 10.3390/ijerph17228581. 33227944 10.3390/ijerph17228581 PMC7699288

[40] Lebel L, Paquin V, Kenny TA, Fletcher C, Nadeau L, Chachamovich E, et al. Climate change and Indigenous mental health in the Circumpolar North: a systematic review to inform clinical practice. Transcult Psychiatry. 2022;59(3):312–36. 34989262 10.1177/13634615211066698 PMC9160950

[41] Pervilhac C, Schoilew K, Znoj H, Müller TJ. [Weather and suicide : association between meteorological variables and suicidal behavior-a systematic qualitative review article]. Nervenarzt. 2020;91(3):227–32. 31468092 10.1007/s00115-019-00795-x

[42] Tito VR, Kazem H, Kadia SO, Paquito B. A systematic review of mental health and climate change in the Philippines. Asian J Psychiatr. 2024;101:104191. 39232390 10.1016/j.ajp.2024.104191

[43] Gao J, Cheng Q, Duan J, Xu Z, Bai L, Zhang Y, et al. Ambient temperature, sunlight duration, and suicide: A systematic review and meta-analysis. Sci Total Environ. 2019;646:1021–9. 30235587 10.1016/j.scitotenv.2018.07.098

[44] Balikuddembe JK, Zheng Y, Prisno DEL 3rd, Stodden R. Impact of climate-induced floods and typhoons on geriatric disabling health among older Chinese and Filipinos: a cross-country systematic review. BMC Geriatr. 2024;24(1):320. 38580910 10.1186/s12877-024-04855-z PMC10998398

[45] Li D, Zhang Y, Li X, Zhang K, Lu Y, Brown RD. Climatic and meteorological exposure and mental and behavioral health: a systematic review and meta-analysis. Sci Total Environ. 2023;892:164435. 37257626 10.1016/j.scitotenv.2023.164435 PMC12919713

[46] Hernández-Duarte WA, Guerrero-Calderon DP, Duarte-Gómez WJ, Fonseca-Paipa AN, Rodríguez-Muñoz M, Tellez-Morales MA, et al. Health conditions in land transport workers and climate change. Exploratory systematic review. Rev Bras Med Trab. 2024;22(2):e20241268. 39371277 10.47626/1679-4435-2024-1268 PMC11452105

[47] Daliri S, Bazyar J, Sayehmiri K, Delpisheh A, Sayehmiri F. The incidence rates of suicide attempts and successful suicides in seven climatic conditions in Iran from 2001 to 2014: a systematic review and meta-analysis. HBI_Journals. 2017;21(6):1.

[48] Doll A, Hölzel BK, Mulej Bratec S, Boucard CC, Xie X, Wohlschläger AM, et al. Mindful attention to breath regulates emotions via increased amygdala-prefrontal cortex connectivity. NeuroImage. 2016;134:305–13. 27033686 10.1016/j.neuroimage.2016.03.041

[49] Calderone A, Latella D, Impellizzeri F, de Pasquale P, Famà F, Quartarone A, et al. Neurobiological changes induced by mindfulness and meditation: a systematic review. Biomedicines. 2024;12(11):2613. 39595177 10.3390/biomedicines12112613 PMC11591838

[50] Vargas-Uricoechea H, Castellanos-Pinedo A, Urrego-Noguera K, Vargas-Sierra HD, Pinzón-Fernández MV, Barceló-Martínez E, et al. Mindfulness-based interventions and the Hypothalamic–Pituitary–Adrenal Axis: a systematic review. Neurol Int. 2024;16(6):1552–84. 39585074 10.3390/neurolint16060115 PMC11587421

[51] Manigault AW, Shorey RC, Hamilton K, Scanlin MC, Woody A, Figueroa WS, et al. Cognitive behavioral therapy, mindfulness, and cortisol habituation: A randomized controlled trial. Psychoneuroendocrinology. 2019;104:276–85. 30917336 10.1016/j.psyneuen.2019.03.009

[52] Talukder B, van Loon GW, Hipel KW, Chiotha S, Orbinski J. Health impacts of climate change on smallholder farmers. One Health. 2021;13:100258. 34027006 10.1016/j.onehlt.2021.100258 PMC8122118

[53] Padhy SK, Sarkar S, Panigrahi M, Paul S. Mental health effects of climate change. Indian J Occup Environ Med. 2015;19(1):3–7. 26023264 10.4103/0019-5278.156997 PMC4446935

[54] Wind TR, Komproe IH. The mechanisms that associate community social capital with post-disaster mental health: a multilevel model. Soc Sci Med. 2012;75(9):1715–20. 22883254 10.1016/j.socscimed.2012.06.032

[55] Wang Z, Zhao Q, Ji Y. The impact of off-farm employment recession and land on farmers’ mental health: empirical evidence from rural China. Land. 2024. 10.3390/land13060837.

[56] Daghagh Yazd S, Wheeler SA, Zuo A. Key risk factors affecting farmers’ mental health: a systematic review. Int J Environ Res Public Health. 2019. 10.3390/ijerph16234849. 31810320 10.3390/ijerph16234849 PMC6926562

[57] Żółtaszek A. Mental and occupational difficulties of the vulnerable groups in the labour force: women, young and older workers, demographic and sexual minorities, and the disabled. Employee Responsib Rights J. 2025. 10.1007/s10672-025-09564-2.

[58] Funari E, Manganelli M, Sinisi L. Impact of climate change on waterborne diseases. Ann Ist Super Sanita. 2012;48(4):473–87. 23247142 10.4415/ANN_12_04_13

[59] Crandon TJ, Dey C, Scott JG, Thomas HJ, Ali S, Charlson FJ. The clinical implications of climate change for mental health. Nat Hum Behav. 2022;6(11):1474–81. 36385181 10.1038/s41562-022-01477-6

[60] Berry HL, Waite TD, Dear KB, Capon AG, Murray V. The case for systems thinking about climate change and mental health. Nat Clim Change. 2018;8(4):282–90.

[61] Thompson R, Lawrance E, Roberts L, Grailey K, Ashrafian H, Maheswaran H, et al. Ambient temperature and mental health: a systematic review and meta-analysis. Lancet Planet Health. 2023;7:e580–9. 37437999 10.1016/S2542-5196(23)00104-3

[62] Gianfredi V, Mazziotta F, Clerici G, Astorri E, Oliani F, Cappellina M, et al. Climate change perception and mental health. Results from a systematic review of the literature. Eur J Investig Health Psychol Educ. 2024;14(1):215–29. 38248134 10.3390/ejihpe14010014 PMC10814599

[63] Okuda K, Kawasaki A. Effects of disaster risk reduction on socio-economic development and poverty reduction. Int J Disaster Risk Reduct. 2022;80:103241.

[64] Tripathi A. Climate Change and Mental Health. Indian J Behav Sci. 2024;27(2).

[65] Militao EMA, Uthman OA, Salvador EM, Vinberg S, Macassa G. Association between socioeconomic position of the household head, food insecurity and psychological health: an application of propensity score matching. BMC Public Health. 2024;24(1):2590. 39334082 10.1186/s12889-024-20153-0 PMC11429249

[66] Murendo C. Exposure to pollution and climate change-induced food insecurity on depressive symptoms among adolescents in rural areas of Afghanistan. Discov Ment Health. 2025;5(1):92. 40537584 10.1007/s44192-025-00172-y PMC12179013

[67] Gasparrini A, Guo Y, Hashizume M, Lavigne E, Zanobetti A, Schwartz J, et al. Mortality risk attributable to high and low ambient temperature: a multicountry observational study. Lancet. 2015;386(9991):369–75. 26003380 10.1016/S0140-6736(14)62114-0 PMC4521077

[68] Schraufnagel DE, Balmes JR, De Matteis S, Hoffman B, Kim WJ, Perez-Padilla R, et al. Health Benefits of Air Pollution Reduction. Ann Am Thorac Soc. 2019;16(12):1478–87. 31774324 10.1513/AnnalsATS.201907-538CME

[69] Gougsa S, Pratt V, Kobei D, Kokunda S, Odochao S, Laiti J, et al. Indigenous mental health research in the context of climate change: methodological reflections on language and barriers to cultural practice. BMJ Ment Health. 2025. 10.1136/bmjment-2025-301856. 40713061 10.1136/bmjment-2025-301856 PMC12306245

[70] Grande AJ, Dias I, Jardim P, Machado A, Soratto J, Rosa M, et al. Climate change and mental health of Indigenous peoples living in their territory: a concept mapping study. Front Psychiatry. 2023. 10.3389/fpsyt.2023.1237740. 38025449 10.3389/fpsyt.2023.1237740 PMC10657843

[71] Faregh N, Lencucha R, Ventevogel P, Dubale BW, Kirmayer LJ. Considering culture, context and community in mhGAP implementation and training: challenges and recommendations from the field. Int J Ment Health Syst. 2019;13:58. 31462908 10.1186/s13033-019-0312-9 PMC6708207

[72] Norris FH, Stevens SP, Pfefferbaum B, Wyche KF, Pfefferbaum. Community Resilience as a Metaphor, Theory, Set of Capacities, and Strategy for Disaster Readiness. Am J Community Psychol. 2008;41:127–50. 18157631 10.1007/s10464-007-9156-6

[73] Kirmayer LJ, Dandeneau S, Marshall E, Phillips MK, Williamson KJ. Rethinking resilience from indigenous perspectives. Can J Psychiatry. 2011;56(2):84–91. 21333035 10.1177/070674371105600203

[74] Du H, Li X, Lin D. Individualism and sociocultural adaptation: discrimination and social capital as moderators among rural-to-urban migrants in China. Asian J Soc Psychol. 2014. 10.1111/ajsp.12085. 10.1111/ajsp.12085 PMC4414335 25937806

[75] Patel V, Saxena S, Lund C, Thornicroft G, Baingana F, Bolton P, et al. The Lancet Commission on global mental health and sustainable development. Lancet. 2018;392(10157):1553–98. 30314863 10.1016/S0140-6736(18)31612-X

[76] Kirmayer L, Sedhev M, Whitley R, Dandeneau S, Isaac C. Community resilience: Models, metaphors and measures. J Aborig Health. 2009;5:62–117.

